# The Effect of Left-Behind Women on Fertilizer Use: Evidence from China’s Rural Households Engaging in Rural-Urban Migration

**DOI:** 10.3390/ijerph20010488

**Published:** 2022-12-28

**Authors:** Kai Tang

**Affiliations:** School of Economics and Trade, Guangdong University of Foreign Studies, Guangzhou 510006, China; francistang1988@hotmail.com

**Keywords:** left-behind women, fertilizer use, smallholder households, rural-urban migration, China

## Abstract

Rural-urban migration in developing countries has required left-behind women to assume the role of key decision makers and take increasing responsibility for agricultural production. However, little is known about the effect of rural-urban migration on fertilizer use when left-behind women assume key decision-maker roles. This study highlights the effect of left-behind women on fertilizer use, drawing on nationwide household survey data in China. The results indicate that households with recognized left-behind women heads use less fertilizer than those with recognized men heads, while households with de facto left-behind women heads use more fertilizer. Moreover, the average nexus between households with recognized left-behind women heads whose major agricultural income comes from grain crops and fertilize use is negative but small in size and statistically insignificant. The findings indicate that future policy efforts aimed at decreasing rural environmental degradation should place greater emphasis on left-behind women’s empowerment in socioeconomic decision-making within and outside the household, thereby contributing to an environment in which left-behind women farmers can succeed in a sustainable way.

## 1. Introduction

In the past few decades many developing countries have experienced enormous rural-urban migration in the process of urbanization and industrialization, which is thought to be one of the key characteristics of those countries’ urban-rural integration [1,2,3,4]. Every year, hundreds of millions of rural residents migrate from rural areas to cities and towns, seeking more and better jobs, higher payments and better amenities [5,6,7,8]. In general, more than half of rural-urban migrants in developing countries are male [3,9,10]. Women’s migration is commonly restricted by socio, economic and cultural norms in many less-developed rural communities [11]. According to the latest rural-urban migration survey report released by the National Bureau of Statistics of China, as of 2021, the number of rural-urban migrants in China, the world’s most populous and the largest developing country, was about 133 million, and more than 64% of the rural-urban migrants were male (http://www.stats.gov.cn/tjsj/zxfb/202204/t20220429_1830126.html, accessed on 13 September 2022). As a result, the absence of men might change prevailing gender power inequalities, showing that men usually play a dominating role in deciding household affairs and require the female left-behind family members to assume the role of key decision makers and take increasing responsibility for agricultural production [12].

In many developing countries where agricultural sectors are characterized as smallholders, the past several decades have witnessed, simultaneously, enormous rural-urban migration and serious environmental problems such as fertilizer overuse. A typical example is China, a country that feeds 22% of the global population on only 7% of the world’s arable land. Due to the increasing population and booming food demand, smallholder farmers significantly increase the use of chemical fertilizer with the purpose of enhancing production [13,14]. However, since the mid-1990s, the increasing rate of chemical fertilizer use has been roughly 77%, 2.3 times the increasing rate of agricultural production, indicating a decreasing efficiency of fertilizer use [15]. According to statistics from the Food and Agriculture Organization of the United Nations, chemical fertilizer use in China was more than 370 kg per hectare, on average, in 2017, while the global average was about 123 kg per hectare [16]. Excessive fertilizer use causes surpluses of nitrogen and phosphate oxide, thus leading to water, soil and air degradation (e.g., surface water eutrophication, soil acidification, and higher greenhouse gas emissions) and threatening human and animal health and biodiversity [17,18,19]. To sustain socioeconomic and ecological systems, it is therefore crucial to understand the drivers and take active measures to reduce fertilizer overuse [20,21,22].

Rural-urban migration in developing countries has attracted increasing attention in the literature. One of the critical issues in the rural-urban migration literature is evaluating the effects. An emerging literature has considered various socioeconomic effects of rural-urban migration in the context of developing countries. These studies have discussed the positive consequences, such as reducing poverty [7,23], increasing opportunities for better education [24,25], better health services [26,27] and improving productivity [28,29] and the negative effects, such as losses of traditional culture [30], weakening social cohesion [31] and raising physical and mental health issues [32,33]. However, the environmental impact of rural-urban migration in developing countries has received far less attention [34,35]. Another critical concern in the rural-urban migration literature is the nexus between rural-urban migration and the environment, which has recently attracted increasing attention [35,36]. The majority of this strand of literature attempts to explore how environmental change (i.e., climate change, environmental degradation such as water scarcity, extreme climate events) shapes rural-urban migration (e.g., [37,38,39,40]). On the contrary, few have been concerned about the effects of rural-urban migration on the rural environment, especially for those developing countries whose rural communities are dominated by smallholder households [41,42]. Very little is known about the effect of rural-urban migration on fertilizer use in developing countries’ smallholder agriculture.

In terms of the sustainable agricultural production literature, existing studies tend to be largely gender-ignored. Only a few studies have highlighted gender inequalities in sustainable agricultural production; women have little control over labor and production and unequal access to resources [43,44,45]. In the context of massive rural-urban migration in developing economies, the migration of men enhances the autonomy of left-behind women in household decision-making. Several studies note that left-behind women have more decision-making power on agricultural production and food security and yet do not consider fertilizer use issues [11,46,47]. Considering that women’s attitudes tend to be more positive toward sustainability [48,49,50], women-headed smallholder households might have an advantage in sustainable agricultural production, such as reducing fertilizer overuse vis-à-vis men-headed smallholder households. Substantial changes in intrahousehold decision-making power status are associated with rural-urban migrating men and left-behind women, including implications for fertilizer use. However, there is limited analysis that empirically explores the effect of rural-urban migration on fertilizer use when left-behind women assume key decision makers.

This study attempts to highlight the effect of left-behind women on fertilizer use by drawing on nationwide survey household data in China and, in doing so, making two main contributions. First, little has been done about the environmental effect of rural-urban migration from the perspective of smallholder households in developing countries. This study empirically analyses the environmental effect of rural-urban migration, with a focus on fertilizer use, using large-scale nationwide survey data covering all provinces in China’s mainland. Second, with a focus on gender social roles change in rural-urban migration, this study provides empirical evidence on how changes in left-behind women’s decision-making roles under conditions of such migration intersect with fertilizer use.

The rest of this study is organized as follows. Section 2 summarizes the data and variables used and presents the empirical strategy used to explore the effects of left-behind women on fertilizer use. Section 3 demonstrates the empirical results. Section 4 discusses, and Section 5 concludes.

## 2. Materials and Methods

### 2.1. Data and Variables Used

**Figure 1:**
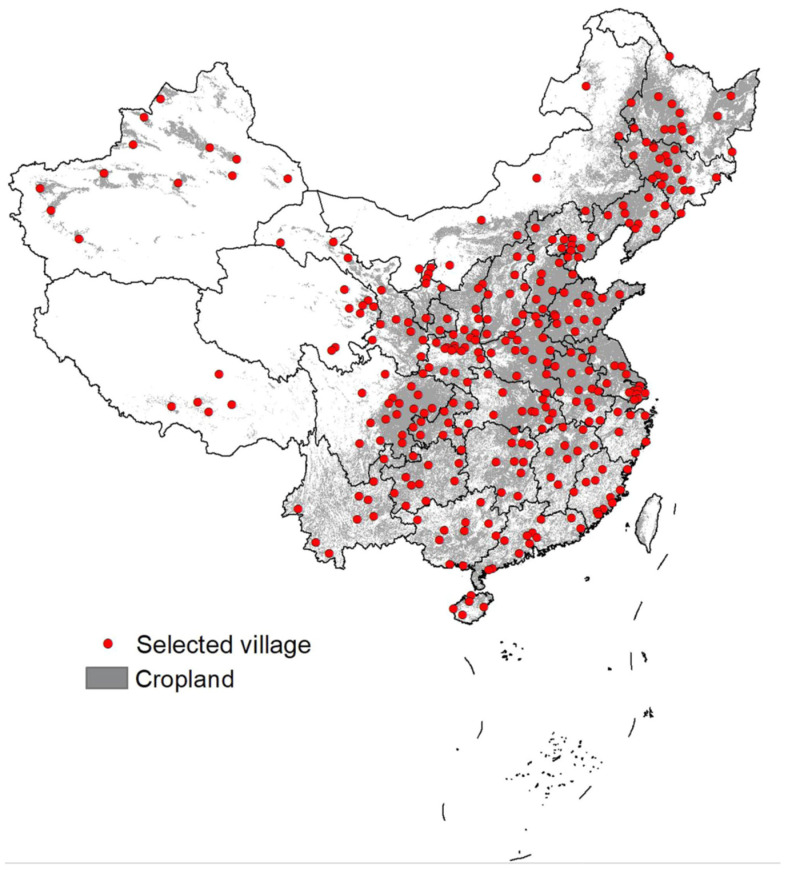
Location of the selected villages for the survey. Source: [50].

The data used are from annual household surveys conducted by the Ministry of Agriculture of China. The survey is a nationwide longitudinal survey of villages and smallholder households that collects detailed household-level information on household characteristics consistently from 360 villages in 357 counties across all 31 provinces and administrative regions in China’s mainland (Figure 1). The sampling method used is a combination of classification sampling and random sampling. The questionnaire used, including more than 900 items in total, covered information such as family status, family members’ migration information, household decisions, and agricultural production and operation activities according to the required household-keeping daily accounts. Further details about the survey can be found in Benjamin et al. (2005) [51] and Gustafsson et al. (2014) [52]. The collected data have been explored in recent literature on various topics in China’s rural communities. See, for example, Benjamin et al. (2011) [53] on inequality and household income growth, Tan et al. (2017) [54] on household consumption, Jin et al. (2021) [50] on household livestock and crop production, and Wang et al. (2022) [55] on farmland abandonment. In this study, the data used are from households engaging in rural-urban migration with full information across all provinces and administrative regions in China’s mainland between 2009 and 2013. Monetary variables are measured using the 2009 constant Chinese yuan with the necessary adjustment based on the official Consumer Price Index (http://www.stats.gov.cn/ztjc/tjzs/cpizzg, accessed on 10 August 2022).

One of the major concerns, the outcome variable in the analysis, is fertilizer use. Due to considerations of data availability and continuity, fertilizer use is measured by the amount of total fertilizer used per unit of cultivated land (kg/mu, 15 mu = 1 hectare). Total fertilizer used is calculated as the sum of nitrogen, phosphate, potassium and compound fertilizers applied.

Another major concern is describing intrahousehold decision-making power status. Considering the enhanced autonomy of left-behind women in intrahousehold decision-making associated with migrating men in the context of massive rural-urban migration, this study creates two binary mitigation outcomes based on the information from the survey: recognized left-behind-woman-headed household and de facto left-behind-woman-headed household. These two binary outcomes are among the predictors identified for fertilizer use.

Recognized left-behind-women-headed households are defined based on China’s household registration (*hukou*) system. Under this system, every China’s citizen is legally bound to register his or her single permanent residential place and the type of *hukou*, either agricultural (rural) or non-agricultural (urban) [56]. The information recorded in the *hukou* system is officially recognized by China’s institutions. Household heads registered in *hukou* systems well reflect their dominance in family decision-making. Since the vast majority of registered household heads are males due to socio, economic and cultural norms [57,58], those households with female registered household heads therefore strongly show female dominance in their family decision-making. In this study, the value of a recognized left-behind-woman-headed household is set as 1 if the studied household has a left-behind female *hukou*-registered household head. Otherwise, the value is set as 0.

A de facto woman-headed household is defined with the consideration of China’s rural-urban migration in the last few decades. In the absence of men who are *hukou*-registered household heads, left-behind women’s decision-making power is likely to substantially increase and sometimes they may become the actual decision-maker. Such a change may have implications for fertilizer use since women’s attitudes tend to be more positive toward sustainability [48,49]. Hence, this study also creates a binary mitigation outcome to reflect the de facto decision-making role of left-behind women. The value of a de facto left-behind-woman-headed household is set as 1 if the studied household with a male *hukou*-registered household head living outside the village more than half a year and his adult female family member staying at home more than half a year annually. Otherwise, the value is set as 0.

The empirical analysis also controls for other compounding factors, including per capita income, per capita production fixed assets, per capita cultivated land, age, education level of the household head, health condition, and the number of household members (Table A1). The existing literature has shown that these factors are also likely to influence smallholder farmers’ fertilizer use [59,60,61,62,63,64,65]. The inclusion of these control variables is helpful in reducing the endogenous effect caused by the omission of key variables in the empirical analysis.

### 2.2. Empirical Strategy

This study employs the random-effects Tobit method to explore the effect of left-behind women on fertilizer use [66,67,68,69]. Compared to the widely used ordinary least squares (OLS) regressions [70,71], the estimation results from the random-effects Tobit method are more robust in dealing with survey data reflecting decisions [72]. In practice, the observed data on the decision about the amount of resources used or dedicated (e.g., in this study, the data for fertilizer use) may contain zero values, which either represent the actual choice of the decision maker (e.g., farmer) or indicate the missing values. Under both circumstances, the random-effects Tobit method, which accommodates both censored observations and within-cluster dependence of the dependent variable, is thought to be more appropriate than OLS regressions since the former addresses issues of selection bias and non-normality and homoscedasticity in residuals distribution [67,73].

Specifically, the random-effects Tobit method is defined as follows:

(1) $$y_{ijt}^{*}=\beta_{0}+\beta_{1}household_{ijt}+\beta_{2}control_{ijt}+\vartheta_{t}+\mu_{j}+\varepsilon_{ijt}\;\mathrm{with}\;\varepsilon_{ijt}\sim N\left(0,\;\sigma^{2}\right),\;\mathrm{with}\;y_{ijt}=y_{ijt}^{*}\;\mathrm{if}\;y_{ijt}^{*}>0,\;\mathrm{and}\;y_{ijt}=0\;\mathrm{otherwise}$$

where $y_{ijt}$ denotes the observed variable of interest, in this study, the amount of total fertilizer used per unit cultivated land, for household *i* in region *j* at year *t*. $y_{ijt}^{*}$ is the latent variable. $household_{ijt}$ are two binary variables employed to describe intrahousehold decision-making power status, including recognized left-behind-women-headed households and de facto left-behind-women-headed households. $control_{ijt}$ denote selected controls. $\vartheta_{t}$ and $\mu_{j}$ represent region- and year-fixed effects, respectively. $\varepsilon_{ijt}$ is the error term. Equation (1) assumes that the effect of $head_{ijt}$ on $y_{ijt}^{*}$ should be monotonic and the distribution of $y_{ijt}^{*}$ is characterized as left-censored.

With the above settings, the estimated $\beta_{1}$ could not be directly compared. After the above random effects Tobit regression, the marginal effect of a regressor $x_{m}$ on the observed $y_{ijt}$, defined as the amount of change in $y_{ijt}$ caused by a unit change in $x_{m}$, is then set as follows:

(2) $$\frac{\partial E[Y_{ijt}|X]}{\partial x_{m}}=\gamma \Phi (y_{ijt}>0)$$

where γ is the estimated coefficient from the random effects Tobit regression. $\Phi (y_{ijt}>0)$ represents the possibility that the observed variable is positive.

## 3. Results

### 3.1. Average Effect of Left-Behind Women on Fertlizer Use

Table 1 reports the main empirical results. The result of the Wald test (χ^2^ = 8309.3) confirms that the null hypothesis that all regression coefficients in the random effects Tobit model equal to zero has been rejected. The estimated value of *ρ* shows that individuals cause 63.2% of the total residual variance.

The average effect on fertilizer use is then reported. In column (1), the estimated coefficient of recognized left-behind-women-headed households is negative (estimated coefficient of −0.565) and statistically significant. As expected, this implies that smallholder households with recognized left-behind women heads use less fertilizer than those with recognized men heads. According to the marginal effect results shown in column (2), fertilizer use is considerably smaller for smallholder households with recognized left-behind women heads. Compared with smallholder households with recognized men heads, those headed by left-behind women use 35.1% less fertilizer.

However, the estimated coefficient of the de facto left-behind-woman-headed household is positive (estimated coefficient of 0.509) at the significance level of 1% (column (1)), which is different from the estimated result of the recognized left-behind-woman-headed household. The estimate indicates that smallholder households with de facto left-behind women heads use more fertilizer. Specifically, they use 31.7% more fertilizer compared with households that are not headed by left-behind women, actually (column (2)).

### 3.2. Considering the Impacts of Grain and Cash Crops on the Effect

Then, this study evaluates whether the effect of left-behind women on fertilizer use varies by crop type. Existing literature has shown that crop types are likely to influence fertilizer use [57,74]. Some grain crops, such as beans, have relatively high nitrogen use efficiency due to their biological nitrogen fixation, substantially reducing the required nitrogen fertilizer application during growth [75,76]. Researchers argue that the average nitrogen use efficiency of beans (80%) is much higher than that of cash crops, such as vegetables and fruits (10%) in China, supporting that cash crops consume more fertilizer than grain crops [57,74]. Therefore, this study considers the possible varied effects of left-behind women on fertilizer use between different groups of smallholder households based on different predominant crop types. Two crop types, grain crops including wheat, rice, corn, beans and tubers and cash crops including vegetables, fruits, cotton, sugar, cigarettes, etc., are integrated in the analysis. Sampled households are categorized as either grain crop type or cash crop type according to their major agricultural income. The results are presented in Table 2.

For smallholder households with recognized left-behind women heads, the estimate for those with major agricultural income coming from grain crops is negative but small in size and statistically insignificant (column (1)). This implies a small, if any, average nexus between households with recognized left-behind women heads and fertilize use. Nevertheless, the estimate for those whose major agricultural income comes from cash crops is statistically significant at the 1% level and negative (estimated coefficient of −0.429), implying a considerable effect on fertilizer use. For smallholder households with de facto left-behind women heads, estimates are both statistically significant and positive, consistently arguing that smallholder households with de facto left-behind women heads use more fertilizer.

### 3.3. Robustness Test

This study also tests the robustness of the main estimates of the effect of left-behind women on fertilizer use. In the main analysis, the random-effects Tobit method is adopted to evaluate the effect. Here, the robustness of the analysis is tested using another widely applied method, the two-part model [77,78]. The two-part model is thought to be appropriate for mixed discrete-continuous outcomes and a robust extension of the conventional Tobit model [78,79,80] and is able to overcome the selection bias issue to some extent [81]. In practice, the two-part model describes the decision-making process in two steps. In the first step, the effect of left-behind women on the probability of using fertilizer is estimated using all observations. In the second step, the effect of left-behind women on the conditional expectation of using fertilizer is evaluated according to observations with fertilizer use larger than zero.

Estimates for both recognized and de facto left-behind-women-headed households are statistically significant. The results in Table 3 consistently indicate that smallholder households with recognized left-behind women heads use less fertilizer than those with recognized men heads, while households with de facto left-behind women heads actually use more fertilizer compared with those not headed by left-behind women, actually. These findings are in line with the above results using the random effects Tobit method, supporting the robustness of the main estimates.

## 4. Discussion

The empirical analysis argues that smallholder households with recognized left-behind women heads use less fertilizer than those with recognized men heads. This can be explained by the observed difference between women and men in terms of attitudes toward sustainability; women tend to have relatively more positive attitudes toward sustainability [48,49]. Women are more likely to nurse private-realm aspects of living with more sustainable behaviors [82,83]. Moreover, family care-taking behaviors and practices are stereotypically done by women, which also extend to sustainable forms of these behaviors being done more often by women. When left-behind women dominate their family decision-making (i.e., a typical example in China is being registered in the *hukou* system as household heads), social stereotypes and a preference to adhere to family care-taking might particularly encourage left-behind women to engage in sustainable behaviors, thus effectively reducing fertilizer use. This also highlights the importance of women’s empowerment in family socioeconomic decision-making.

The empirical analysis shows that smallholder households with de facto left-behind women heads use more fertilizer. In fact, left women’s de facto household headship well reflects women’s shift from assistants to main functionaries in family affairs [47], not certainly ensuring the change of left-behind women’s dependence upon migrating male spouses. The majority of left-behind women still regard the men who have been registered as the household heads in the *hukou* system as de jure household heads. Migrating male household heads are commonly consulted before making any major household decisions, showing the incomplete decision-making power and autonomy of left-behind women. Studies have shown that men’s engagement in sustainable activities tends to be circumscribed due to social stereotypes and a preference for adhering to gender role norms [83]. Men are more likely to avoid or even oppose sustainable activities for the purpose of safeguarding their gender identity with consideration of the prevalent connection between the concepts of sustainability and femininity (the green-feminine stereotype) [84,85]. Under such circumstances, the potentially sustainable effects of women’s enhanced autonomy are limited.

Moreover, the gender-based disadvantages suffered by left-behind women may further undermine the sustainable effects. Rural women usually have less scientific knowledge than men due to rural women’s limited access to education in developing countries [47,86]. Households with de facto women heads also have relative disadvantages in accessing government-run agricultural technology promotion programs, considering that officially registered male household heads are absent. Therefore, the fertilizer use decision of those households with de facto left-behind women heads might not be appropriate, resulting in relatively higher fertilizer use.

The empirical results show that the average nexus between households with recognized left-behind women heads whose major agricultural income comes from grain crops and fertilize use is negative but small in size and statistically insignificant. In some developing counties, including China, the production of grain crops is usually thought to be less profitable than cash crops in the smallholder agricultural sector [87,88]. In China, due to the application of strict grain price regulations since the late 1990s [89], the profitability gap between grain crop production and cash crop production might be even more evident. Farmers enjoy additional profits through producing more organic cash crops (e.g., fruits and vegetables) with less fertilizer use since those cash crops are likely to have considerably higher prices than those produced in a conventional fertilizer-intensive way. However, famers in China are less likely to gain similar additional profits through producing grain crops with less fertilizer use because the market prices of grain commodities are heavily influenced by the government’s grain price regulations and other policies. Consequently, it is difficult for recognized left-behind-women-headed households with major agricultural income coming from grain crops to maintain profitability with less fertilizer use, which may further result in a small and insignificant estimate.

## 5. Conclusions

This study has explored the potential link between two observed recent trends in China: left-behind women’s rising autonomy in household decision-making accompanying massive rural-urban migration and increasing fertilizer use in agricultural production. Drawing on nationwide survey household data, this study highlights the effect of left-behind women on fertilizer use, which has rarely been considered in the existing literature.

The findings show that in the context of China, households with recognized left-behind women heads use 35.1% less fertilizer than those with recognized men heads. However, households with de facto left-behind women heads use 31.7% more fertilizer compared with those not headed by left-behind women, actually. Furthermore, the average effect of households with recognized left-behind women heads whose major agricultural income comes from grain crops on fertilize use is negative but small in size and statistically insignificant.

The results suggest that future policy efforts aimed at decreasing rural environmental degradation should place greater emphasis on left-behind women’s empowerment in socioeconomic decision-making within and outside the household. The growing trend of the feminization of agriculture in the developing world should be encouraged, as it is essential to enhance left-women’s autonomy to further sustain rural communities. Policies and institutions need to ensure left-behind women’s equal access to public agricultural technology promotion programs and financial incentives, thus contributing to an environment in which left-behind women farmers can succeed in a sustainable way.

This study, however, has several limitations. Due to data unavailability, the analysis only considers the amount of total fertilizer used, rather than the amount of specific fertilizer (e.g., nitrogen, phosphate, potassium and compound fertilizers). A more detailed analysis based on the use of these fertilizers separately would be helpful in the future. Additionally, a comparison using data from different developing countries would also be needed to better understand the effect of left-behind women on fertilizer use in the developing context.

## Figures and Tables

**Table 1:** Average impacts of left-behind women on fertilizer use.

Explained Variable: Fertilizer Used per Unit of Cultivated Land		
Model	(1)	(2)
Method	Random Effects Tobit	Marginal Effect
Recognized left-behind-woman-headed household	−0.565 ***	−0.351 ***
	(0.066)	(0.041)
De facto left-behind-woman-headed household	0.509 ***	0.317 ***
	(0.049)	(0.030)
Control variables	YES	YES
Region-fixed effect	YES	YES
Year-fixed effect	YES	YES
Constant	−1.276 ***	--
	(0.395)	--
*σ_μ_*	2.556 (0.017)	--
*σ_e_*	1.953 (0.006)	--
*ρ*	0.632 (0.004)	--
Log likelihood	−168,447.72	--
Wald test (χ^2^)	8309.30	--

Note: Fertilizer use and several controls including per capita income, per capita production fixed assets and per capita cultivated land are in log form. *** denotes significance levels of 1%. Standard errors are in parentheses.

**Table 2:** Impacts of left-behind women on fertilizer use with different crop types.

Explained Variable: Fertilizer Used per Unit of Cultivated Land		
Model	(1)	(2)
Sample	Grain Crops	Cash Crop
Method	Random Effects Tobit	Random Effects Tobit
Recognized left-behind-woman-headed household	−0.006	−0.429 ***
	(0.037)	(0.096)
De facto left-behind-woman-headed household	0.083 ***	0.147 *
	(0.030)	(0.077)
Control variables	YES	YES
Region-fixed effect	YES	YES
Year-fixed effect	YES	YES
Constant	1.235 ***	−2.946 ***
	(0.163)	(0.685)
*σ_μ_*	0.901(0.009)	1.942(0.024)
*σ_e_*	1.027(0.004)	1.711(0.011)
*ρ*	0.435(0.006)	0.563(0.007)
Log likelihood	−78,458.452	−46,282.046
Wald test (χ^2^)	3002.97	2177.40

Note: Fertilizer use and several controls including per capita income, per capita production fixed assets and per capita cultivated land are in log form. *** and * denote significance levels of 1% and 10%, respectively. Standard errors are in parentheses.

**Table 3:** Results of robustness test: two-part model.

Explained Variable: Fertilizer Used per Unit Cultivated Land			
Model	(1)	(2)	(3)
	Part One		Part Two
	Coefficient	Marginal Effect	Coefficient
Recognized left-behind-woman-headed household	−0.379 ***	−0.013 ***	−0.099 ***
	(0.068)	(0.002)	(0.018)
De facto left-behind-woman-headed household	0.425 ***	0.014 ***	0.021 *
	(0.075)	(0.003)	(0.013)
Control variables	YES	YES	YES
Region-fixed effect	YES	YES	YES
Year-fixed effect	YES	YES	YES
Constant	−0.087	--	3.734 ***
	(0.276)	--	(0.112)
*σ_μ_*	1.903(0.039)	--	0.696(0.004)
*σ_e_*	--	--	0.481(0.002)
*ρ*	0.784(0.007)	--	0.676(0.003)
Log likelihood	−18,309.9	--	−65,454.047
Wald test (χ^2^)	3907.56	--	--

Note: Fertilizer use and several controls including per capita income, per capita production fixed assets and per capita cultivated land are in log form. *** and * denote significance levels of 1% and 10%, respectively. Standard errors are in parentheses.

## Data Availability

Restrictions apply to the availability of these data. Data was obtained from the Ministry of Agriculture of China and are available from the author with the permission of the Ministry of Agriculture of China.

## Appendix A

**Table A1:** Variables and statistics.

Variable	Definition	Mean	Std. Dev.
Fertilizer use	The amount of total fertilizer used per unit cultivated land (kg/mu ^a^)	486.3	120.5
Recognized left-behind-woman-headed household	*Hukou*-registered household head is female = 1; otherwise = 0	0.07	0.03
De facto left-behind-woman-headed household	Household with a male *hukou*-registered head living outside village more than half year and his adult female family member staying at home more than half year annually = 1; otherwise = 0	0.20	0.04
Income	Per capital income (yuan)	31,162	13,058
Production fixed assets	Per capital production fixed assets (yuan)	19,247	2871
Cultivated land	Per capital cultivated land area (mu)	2.14	1.04
Age	Years	43.33	14.04
Education level of household head	Education years of household head	6.90	2.60
Health condition	Self-reported health condition; disabled = 1, bad = 2, normal = 3, good = 4, excellent = 5 ^b^	4.07	1.31
Number of household members	Number of family members	3.92	1.64

Note: ^a^ 15 mu = 1 hectare; ^b^ Self-reported health condition measured from 1 (disabled) to 5 (excellent).

## References

[1] Ge D., Long H., Qiao W., Wang Z., Sun D., Yang R. (2020). Effects of rural–urban migration on agricultural transformation: A case of Yucheng City, China. J. Rural Stud..

[2] Imbert C., Papp J. (2020). Costs and benefits of rural-urban migration: Evidence from India. J. Dev. Econ..

[3] Selod H., Shilpi F. (2021). Rural-urban migration in developing countries: Lessons from the literature. Reg. Sci. Urban Econ..

[4] Tang K., Liu Y., Zhou D., Qiu Y. (2021). Urban carbon emission intensity under emission trading system in a developing economy: Evidence from 273 Chinese cities. Environ. Sci. Pollut. Res..

[5] Xing C., Zhang J. (2017). The preference for larger cities in China: Evidence from rural-urban migrants. China Econ. Rev..

[6] Wang Z., Chen L. (2019). Destination choices of Chinese rural–urban migrant workers: Jobs, amenities, and local spillovers. J. Reg. Sci..

[7] Abeje A. (2021). Causes and effects of rural-urban migration in Ethiopia: A case study from Amhara Region. Afr. Stud..

[8] Choithani C., van Duijne R.J., Nijman J. (2021). Changing livelihoods at India’s rural–urban transition. World Dev..

[9] Chen C., Fan C.C. (2018). Rural-urban circularity in China: Analysis of longitudinal surveys in Anhui, 1980–2009. Geoforum.

[10] Hidrobo M., Mueller V., Roy S. (2022). Cash transfers, migration, and gender norms. Am. J. Agric. Econ..

[11] Choithani C. (2020). Gendered livelihoods: Migrating men, left-behind women and household food security in India. Gend. Place Cult..

[12] Ye J., Wu H., Rao J., Ding B., Zhang K. (2016). Left-behind women: Gender exclusion and inequality in rural-urban migration in China. J. Peasant Stud..

[13] Tang K., Ma C. (2022). The cost-effectiveness of agricultural greenhouse gas reduction under diverse carbon policies in China. China Agric. Econ. Rev..

[14] Tang K., Hailu A. (2020). Smallholder farms’ adaptation to the impacts of climate change: Evidence from China’s Loess Plateau. Land Use Policy.

[15] National Statistical Bureau of China (2021). China Statistical Yearbook.

[16] FAO, FAOSTAT FAO Statistical Databases, Rome, Italy, 2019. http://www.fao.org/faostat/en/#home/.

[17] Tang K., Gong C., Wang D. (2016). Reduction potential, shadow prices, and pollution costs of agricultural pollutants in China. Sci. Total Environ..

[18] Tang K., Hailu A., Yang Y. (2020). Agricultural chemical oxygen demand mitigation under various policies in China: A scenario analysis. J. Clean. Prod..

[19] Van Wesenbeeck C.F.A., Keyzer M.A., Van Veen W.C.M., Qiu H. (2021). Can China’s overuse of fertilizer be reduced without threatening food security and farm incomes?. Agric. Syst..

[20] Tang K., Hailu A., Kragt M.E., Ma C. (2016). Marginal abatement costs of greenhouse gas emissions: Broadacre farming in the Great Southern Region of Western Australia. Aust. J. Agric. Resour. Econ..

[21] Tang K., Kragt M.E., Hailu A., Ma C. (2016). Carbon farming economics: What have we learned?. J. Environ. Manag..

[22] Tang K., Wang M., Zhou D. (2021). Abatement potential and cost of agricultural greenhouse gases in Australian dryland farming system. Environ. Sci. Pollut. Res..

[23] Ingelaere B., Christiaensen L., De Weerdt J., Kanbur R. (2018). Why secondary towns can be important for poverty reduction–A migrant perspective. World Dev..

[24] Nguyen L.D., Raabe K., Grote U. (2015). Rural–urban migration, household vulnerability, and welfare in Vietnam. World Dev..

[25] Amrevurayire E.O., Ojeh V.N. (2016). Consequences of rural-urban migration on the source region of Ughievwen clan Delta State Nigeria. Eur. J. Geogr..

[26] Cheng Y., Rosenberg M., Winterton R., Blackberry I., Gao S. (2019). Mobilities of older Chinese rural-urban migrants: A case study in Beijing. Int. J. Environ. Res. Public Health.

[27] Shi X. (2022). Moving out but not for the better: Health consequences of interprovincial rural-urban migration in China. Health Econ..

[28] Combes P.P., Démurger S., Li S. (2017). Productivity gains from agglomeration and migration in the People’s Republic of China between 2002 and 2013. Asian Dev. Rev..

[29] Bryan G., Morten M. (2019). The aggregate productivity effects of internal migration: Evidence from Indonesia. J. Polit. Econ..

[30] Liu Y., Yang Y., Li Y., Li J. (2017). Conversion from rural settlements and arable land under rapid urbanization in Beijing during 1985–2010. J. Rural Stud..

[31] King M., Smith A., Gracey M. (2009). Indigenous health part 2: The underlying causes of the health gap. Lancet.

[32] Mao Z.H., Zhao X.D. (2012). The effects of social connections on self-rated physical and mental health among internal migrant and local adolescents in Shanghai, China. BMC Public Health.

[33] Li J., Rose N. (2017). Urban social exclusion and mental health of China’s rural-urban migrants–A review and call for research. Health Place.

[34] Robson J.P., Berkes F. (2011). Exploring some of the myths of land use change: Can rural to urban migration drive declines in biodiversity?. Glob. Environ. Chang..

[35] Greiner C., Sakdapolrak P. (2013). Rural–urban migration, agrarian change, and the environment in Kenya: A critical review of the literature. Popul. Environ..

[36] Millock K. (2015). Migration and environment. Annu. Rev. Resour. Econ..

[37] Marchiori L., Maystadt J.F., Schumacher I. (2012). The impact of weather anomalies on migration in sub-Saharan Africa. J. Environ. Econ. Manag..

[38] Ishtiaque A., Nazem N.I. (2017). Household-level disaster-induced losses and rural–urban migration: Experience from world’s one of the most disaster-affected countries. Nat. Hazards.

[39] Weinreb A., Stecklov G., Arslan A. (2020). Effects of changes in rainfall and temperature on age-and sex-specific patterns of rural-urban migration in sub-Saharan Africa. Popul. Environ..

[40] Mianabadi A., Davary K., Kolahi M., Fisher J. (2022). Water/climate nexus environmental rural-urban migration and coping strategies. J. Environ. Plan. Manag..

[41] Qin H. (2010). Rural-to-urban labor migration, household livelihoods, and the rural environment in Chongqing Municipality, Southwest China. Hum. Ecol..

[42] Li S., Sun Z., Tan M., Li X. (2016). Effects of rural–urban migration on vegetation greenness in fragile areas: A case study of Inner Mongolia in China. J. Geogr. Sci..

[43] Chikwendu D.O., Arokoyo J.O. (1997). Women and sustainable agricultural development in Nigeria. J. Sustain. Agric..

[44] Bahta Y.T., Strydom D.B., Donkor E. (2017). Microcredit and gender empowerment: Policy implications for sustainable agricultural development in Eritrea. Dev. Pract..

[45] Uduji J.I., Okolo-Obasi E.N., Asongu S.A. (2019). Corporate social responsibility and the role of rural women in sustainable agricultural development in sub-Saharan Africa: Evidence from the Niger Delta in Nigeria. Sustain. Dev..

[46] Maharjan A., Bauer S., Knerr B. (2012). Do rural women who stay behind benefit from male out-migration? A case study in the hills of Nepal. Gend. Technol. Dev..

[47] Wu H., Ye J. (2016). Hollow lives: Women left behind in rural China. J. Agrar. Chang..

[48] Karami E., Mansoorabadi A. (2008). Sustainable agricultural attitudes and behaviors: A gender analysis of Iranian farmers. Environ. Dev. Sustain..

[49] Bhattacharyya A., Rahman M.L. (2020). Values, gender and attitudes towards environmental policy: A study of future managers. Bus. Strategy Environ..

[50] Jin S., Zhang B., Wu B., Han D., Hu Y., Ren C., Zhang C., Wei X., Wu Y., Mol A.P. (2021). Decoupling livestock and crop production at the household level in China. Nat. Sustain..

[51] Benjamin D., Brandt L., Giles J. (2005). The evolution of income inequality in rural China. Econ. Dev. Cult. Chang..

[52] Gustafsson B., Shi L., Sato H. (2014). Data for studying earnings, the distribution of household income and poverty in China. China Econ. Rev..

[53] Benjamin D., Brandt L., Giles J. (2011). Did higher inequality impede growth in rural China?. Econ. J..

[54] Tan T., Zhang Y., Wen B., Zhang X., Zhan J. (2017). Household consumption distribution in rural China: A consistent two-step estimation. Can. J. Agric. Econ..

[55] Wang J., Yu C.A.O., Fang X., Li G., Cao Y. (2022). Does land tenure fragmentation aggravate farmland abandonment? Evidence from big survey data in rural China. J. Rural Stud..

[56] Afridi F., Li S.X., Ren Y. (2015). Social identity and inequality: The impact of China’s hukou system. J. Public Econ..

[57] Zhang X., Davidson E.A., Mauzerall D.L., Searchinger T.D., Dumas P., Shen Y. (2015). Managing nitrogen for sustainable development. Nature.

[58] Tang J., Xu Y., Qiu H. (2022). Integration of migrants in poverty alleviation resettlement to urban China. Cities.

[59] Odegard I.Y.R., van der Voet E. (2014). The future of food—Scenarios and the effect on natural resource use in agriculture in 2050. Ecol. Econ..

[60] Bai X., Zhang T., Tian S. (2020). Evaluating fertilizer use efficiency and spatial correlation of its determinants in China: A geographically weighted regression approach. Int. J. Environ. Res. Public Health.

[61] Yuan F., Tang K., Shi Q. (2021). Does Internet use reduce chemical fertilizer use? Evidence from rural households in China. Environ. Sci. Pollut. Res..

[62] Yuan F., Tang K., Shi Q., Qiu W., Wang M. (2022). Rural women and chemical fertiliser use in rural China. J. Clean. Prod..

[63] Zhang C., Sun Y., Hu R., Yang F., Shen X. (2021). The impact of rural-urban migration experience on fertilizer use: Evidence from rice production in China. J. Clean. Prod..

[64] Wu J., Xu H., Tang K. (2021). Industrial agglomeration, CO_2_ emissions and regional development programs: A decomposition analysis based on 286 Chinese cities. Energy.

[65] Wu J., Feng Z., Tang K. (2021). The dynamics and drivers of environmental performance in Chinese cities: A decomposition analysis. Environ. Sci. Pollut. Res..

[66] Brown S., Greene W.H., Harris M.N., Taylor K. (2015). An inverse hyperbolic sine heteroskedastic latent class panel tobit model: An application to modelling charitable donations. Econ. Model..

[67] Amore M.D., Murtinu S. (2021). Tobit models in strategy research: Critical issues and applications. Glob. Strategy J..

[68] Athari S.A. (2021). The effects of institutional settings and risks on bank dividend policy in an emerging market: Evidence from Tobit model. Int. J. Financ. Econ..

[69] Yu Y., Tang K. (2023). Does financial inclusion improve energy efficiency?. Technol. Forecast. Soc. Chang..

[70] Chen X., Chen G., Lin M., Tang K., Ye B. (2022). How does anti-corruption affect enterprise green innovation in China’s energy-intensive industries?. Environ. Geochem. Health.

[71] Tang K., Chen Q., Tan W., Wu Feng Y.J. (2022). The impact of financial deepening on carbon reductions in China: Evidence from city-and enterprise-level data. Int. J. Environ. Res. Public Health.

[72] Greene W.H. (2012). Econometric Analysis.

[73] Wang W., Griswold M.E. (2016). Estimating overall exposure effects for the clustered and censored outcome using random effect Tobit regression models. Stat. Med..

[74] Ren C., Jin S., Wu Y., Zhang B., Kanter D., Wu B., Xi X., Zhang X., Chen D., Xu J. (2021). Fertilizer overuse in Chinese smallholders due to lack of fixed inputs. J. Environ. Manag..

[75] Tang K., Hailu A., Kragt M.E., Ma C. (2018). The response of broadacre mixed crop-livestock farmers to agricultural greenhouse gas abatement incentives. Agric. Syst..

[76] Tang K., He C., Ma C., Wang D. (2019). Does carbon farming provide a cost-effective option to mitigate GHG emissions? Evidence from China. Aust. J. Agric. Resour. Econ..

[77] Madden D. (2008). Sample selection versus two-part models revisited: The case of female smoking and drinking. J. Health Econ..

[78] Belotti F., Deb P., Manning W.G., Norton E.C. (2015). Twopm: Two-part models. Stata J..

[79] Smith V.A., Preisser J.S., Neelon B., Maciejewski M.L. (2014). A marginalized two-part model for semicontinuous data. Stat. Med..

[80] Smith V.A., Preisser J.S. (2019). A marginalized two-part model with heterogeneous variance for semicontinuous data. Stat. Methods Med. Res..

[81] Bushway S., Johnson B.D., Slocum L.A. (2007). Is the magic still there? The use of the Heckman Two-Step Correction for selection bias in Criminology. J. Quant. Criminol..

[82] Bulut Z.A., Kökalan Çımrin F., Doğan O. (2017). Gender, generation and sustainable consumption: Exploring the behaviour of consumers from Izmir, Turkey. Int. J. Consum. Stud..

[83] Bloodhart B., Swim J.K. (2020). Sustainability and consumption: What’s gender got to do with it?. J. Soc. Issues.

[84] Brough A.R., Wilkie J.E., Ma J., Isaac M.S., Gal D. (2016). Is eco-friendly unmanly? The green-feminine stereotype and its effect on sustainable consumption. J. Consum. Res..

[85] Pinna M. (2020). Do gender identities of femininity and masculinity affect the intention to buy ethical products?. Psychol. Mark..

[86] Qiu P., Caine E.D., Hou F., Cerulli C., Wittink M.N. (2018). Depression as seen through the eyes of rural Chinese women: Implications for help-seeking and the future of mental health care in China. J. Affect. Disord..

[87] Yu B., Liu F., You L. (2012). Dynamic agricultural supply response under economic transformation: A case study of Henan, China. Am. J. Agric. Econ..

[88] Li L., Wang C., Segarra E., Nan Z. (2013). Migration, remittances, and agricultural productivity in small farming systems in Northwest China. China Agric. Econ. Rev..

[89] Selim S. (2015). The impact of grain self-sufficiency regime on regional welfare and agricultural productivity in China. Agric. Econ..

